# Visualization of conformational transition of GRP94 in solution

**DOI:** 10.26508/lsa.202302051

**Published:** 2023-11-13

**Authors:** Shangwu Sun, Rui Zhu, Mengyao Zhu, Qi Wang, Na Li, Bei Yang

**Affiliations:** 1 Shanghai Institute for Advanced Immunochemical Studies and School of Life Science and Technology, ShanghaiTech University , Shanghai, China; 2 National Facility for Protein Science in Shanghai, Shanghai Advanced Research Institute (Zhangjiang Laboratory), Chinese Academy of Sciences, Shanghai, China; 3 Shanghai Frontiers Science Center for Biomacromolecules and Precision Medicine, ShanghaiTech University , Shanghai, China; 4 Shanghai Clinical Research and Trial Center, Shanghai, China

## Abstract

GRP94, an ER paralog of the heat-shock protein 90 family, binds and hydrolyses ATP to chaperone the folding and maturation of its selected clients. Compared with other hsp90 proteins, the in-solution conformational dynamics of GRP94 along the ATP hydrolysis cycle are less understood, hindering our understanding of its chaperoning mechanism. Leveraging small-angle X-ray scattering, negative-staining EM, and hydrogen–deuterium exchange coupled mass-spec, here we show that in its apo form, ∼60% of mouse GRP94 (mGRP94) populates an “extended” conformation, whereas the rest exist in either “close V” or “twist V” like “compact” conformations. Different from other hsp90 proteins, the presence of AMPPNP only impacts the relative abundance of the two compact conformations, rather than shifting the equilibrium between the “extended” and “compact” conformations of mGRP94. HDX-MS study of apo, AMPPNP-bound, and ADP-bound mGRP94 suggests a conformational transition from “twist V” to “close V” upon ATP binding and a back transition from “close V” to “twist V” upon ATP hydrolysis. These results illustrate the dissimilarities of GRP94 in conformation transition during ATP hydrolysis from other hsp90 paralogs.

## Abstract (precis)

SAXS, nsEM, and HDX-MS reveal that one extended and two compact conformations of GRP94 coexist in solution and that the binding and hydrolysis of ATP only drives the transition between the two compact conformations while leaving the extended population unaffected.

## Introduction

Glucose-regulated protein of 94 kD (GRP94 or Gp96) is an ER paralog of the heat-shock protein 90 family (hsp90) that binds and hydrolyses ATP to chaperone the folding and maturation of its clients (Lee et al, 1981; Wu et al, 2016). It is present in most multicellular eukaryotes and appears obligatory in the early developmental stage of metazoans (Marzec et al, 2012). Interestingly, up-regulated expression of GRP94 was often observed in advanced stages of various cancers and correlates with poor prognosis (Ansa-Addo et al, 2016). Different from its cytosolic paralogs Hsp90α and Hsp90β which govern the folding and activation of a wide array of proteins, the client list of GRP94 remains highly selective to receptors and secretory proteins (Marzec et al, 2012), including some cancer-associated clienteles like Wnt co-receptor LRP6 (Liu et al, 2013) and HER2 (Patel et al, 2013).

GRP94 generally exists as obligatory dimers with each monomer comprising three major domains: N-terminal (N), middle (M), and C-terminal (C) dimerization domains (Dollins et al, 2007). Like other hsp90s, the N and M domains of GRP94 are involved in nucleotide binding and ATP hydrolysis, whereas the C domain provides the main dimerization interface (Dollins et al, 2007). The N and M domains of GRP94 are connected by a charged linker (CL) region, which likely regulates the chaperone function of GRP94 (Hainzl et al, 2009; Tsutsumi et al, 2009; Tsutsumi et al, 2012; Jahn et al, 2014).

In higher eukaryotes, GRP94 has three paralogs, including the cytosolic Hsp90 α/β and the mitochondrial TRAP1 (Johnson, 2012). Thus far, cytosolic hsp90s have been characterized extensively. Structural studies of cytosolic Hsp90 and its orthologs HtpG (*E.coli*) and hsc82 (*S.cerevisia*e) in different nucleotide, co-chaperone, and/or clientele loaded states together revealed that the N, M, and C domains of cytosolic hsp90s undergo relative rigid-body movements upon nucleotide binding, suggesting a nucleotide-induced cycle of conformational rearrangements that likely correlates to their chaperone functions (Ali et al, 2006; Shiau et al, 2006; Verba et al, 2016). In the absence of nucleotide, the crystal structure of the HtpG dimer adopts an “open V” conformation wherein its two N domains are 150 Å apart from each other (Shiau et al, 2006). The presence of AMPPNP, a non-hydrolyzable analog of ATP, induces the transition of HtpG dimer into a “closed V” shape (Southworth & Agard, 2008) that closely resembles the structure of yeast hsc82 in complex with AMPPNP and a co-chaperone p23 (Ali et al, 2006). More recently, the structure of human Hsp90β trapping an unfolded client was further revealed by cryo-EM, providing the first visualization of cytosolic hsp90 at work (Verba et al, 2016). Interestingly, although crystallographic studies of cytosolic hsp90s revealed distinct conformations of them under nucleotide-free and AMPPNP-bound state, small-angle X-ray scattering (SAXS) analysis, single-molecule FRET, and nsEM reconstitutions rather indicate that multiple conformations of cytosolic hsp90s, for example, “open V” and “close V”, coexist in a dynamic equilibrium under different nucleotide-bound states, where the presence of nucleotide biases the equilibrium rather than triggering a discrete conformational change (Bron et al, 2008; Krukenberg et al, 2008; Southworth & Agard, 2008; Krukenberg et al, 2009; Mickler et al, 2009; Huang et al, 2019). Meanwhile, how far the nucleotide binding shifts the positioning of the equilibria between different conformations appears homolog-specific, as the presence of nucleotide seems to play a more deterministic role in HtpG and hsc82 than it does in mammalian Hsp90α (Southworth & Agard, 2008).

Although similarities are obvious, the differences between GRP94 and cytosolic hsp90s are also prominent. The ATP hydrolysis rate of different hsp90s varies significantly from each other, and GRP94 is much less potent in ATP turnover than hsc82 or HtpG (Dollins et al, 2007; Que et al, 2018). Meanwhile, although the structural model of GRP94 in a nucleotide-free state has not been characterized, structures of canine GRP94 (dGRP94) in complex with AMPPNP or ADP are available, which manifest dissimilarities from other hsp90s despite the highly analogous structures of their individual N, M or C domains. In the presence of AMPPNP, dGRP94 has been captured in both “twisted V” and “closed V” conformations, with the former conformation being unique to GRP94 (Dollins et al, 2007; Huck et al, 2017). In the “twisted V” conformation, the two N domains are close to each other yet oriented in opposing directions, thereby preventing the dimerization of the N domains that are obligatory for its ATPase activity (Dollins et al, 2007); whereas the “closed V” conformation of dGRP94 is similar to that observed in hsc82-AMPPNP-p23 complex, thus representing a ATP hydrolysis competent state of GRP94 (Huck et al, 2017). Interestingly, the “closed V” conformation was only observed when the pre-N domain (22–72 aa, the sequence before the N domain) of dGRP94 was maintained at a minimum length (48–72 aa), suggesting an important role of pre-N domain in regulating the ATPase activity of GRP94. Consistent with this notion, the pre-N domain is conserved in GRP94 and appears to be much longer than its equivalents in other hsp90s, and its deletion considerably up-regulates the ATPase activity of GRP94 (Dollins et al, 2007; Huck et al, 2017).

Compared with our knowledge about the cytosolic hsp90s, GRP94 remains an enigma. To date, it is still not clear how it recognizes its client proteins and how it bridges its ATP hydrolysis activity to the folding of its clients. To resolve these remaining challenges of GRP94, it is important to fully understand its conformational transitions along the ATP hydrolysis cycle, ideally in physiologically relevant solution conditions. nsEM, SAXS, and hydrogen–deuterium eXchange-coupled mass spectrometer (HDX-MS) are powerful and complementary tools to monitor the native conformations of macromolecules in solution at either the global or peptide level. Here, we combined these tools to probe the conformational transitions of mouse GRP94 (mGRP94) in different nucleotide-bound states and revealed the in-solution dynamics of GRP94 along the ATP cycle. This study thus provides information for future investigations of how the conformational changes of GRP94 are relayed to the folding of its clients.

## Results

### apo mGRP94 exists as a heterogenous population wherein extended conformations are present

**Figure S1.**
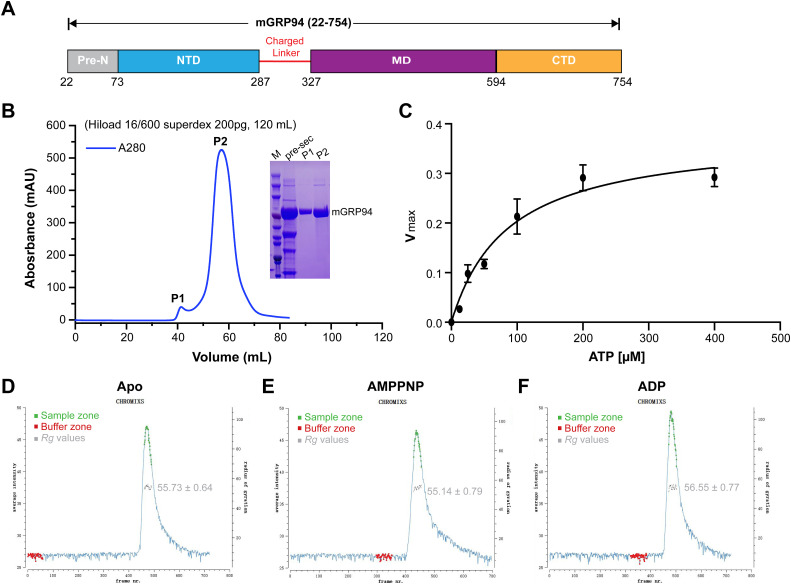
The inline SEC-SAXS profiles of mGRP94 in different states. **(A)** Schematic diagram illustrating the construct of mGRP94 (22–754 aa) with the Pre-N and charged linker regions preserved. Pre-N, pre-N terminal domain; NTD, N-terminal domain; MD, middle domain; CTD, C-terminal domain. **(B)** Gel filtration profiles of purified mGRP94. Shown in the inset are the SDS–PAGE results of indicated peak fractions. **(C)** ATPase activity of purified mGRP94 presented as Michaelis–Menten plot. Experiments were independently performed three times and error bars depict the standard error of the mean. **(D, E, F)** The inline SEC-SAXS profiles of mGRP94 in the absence of nucleotides (D) and in the presence of AMPPNP (E) or ADP (F). The *Rg*s of the samples are also shown.

**Figure 1.**
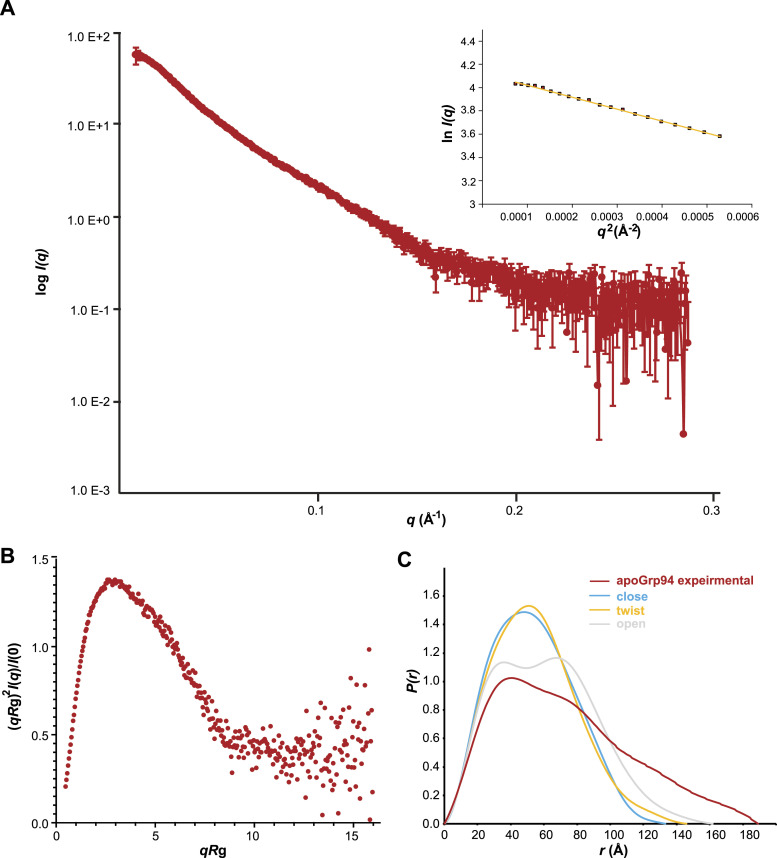
Apo mGRP94 samples extended conformation in solution. **(A)** *I(q)* versus *q* as log-linear plots, the inset shows the Guinier fits (yellow lines) for determination of *R*g. The red squares indicate data within the Guinier region with *qR*g < 1.3. **(A, B)** Dimensionless Kratky plots calculated from the scattering data in (A). **(C)** Normalized *P(r)* curves simulated from the structural models of mGRP94 in “open V” (grey), “close V” (cyan) or “twist V” (yellow) conformation are compared with the normalized experimental *P(r)* of mGRP94 in apo form (red). Neither of the structural models matches the experimental SAXS data of mGRP94 in apo form. The *P(r)* curves are normalized to equal areas.

As an initial step to characterize the physiological dynamics of GPR94 in its apo form, we performed inline size-exclusion chromatography (SEC)-SAXS on purified mouse GRP94 (mGRP94) in the absence of nucleotides (Fig S1A–D). Notably, we preserved the CL region and the full pre-N domain in the mGRP94 construct in consideration of their regulatory role in modulating the GRP94 function (Fig S1A). The scattering intensity, I(*q*), was measured with scattering vectors (*q* = 4πsinθ/λ) ranging from 0.009 to 0.29 Å^−1^ (Fig 1A), and the *R*g for apo mGRP94 was calculated to be 55.7 Å after the Guinier approximation (Fig 1A, inset and Table S1). Dimensionless Kratky plot indicates that apo mGRP94 is largely well folded, despite that the small rise at *qR*g > 10 suggests the presence of minor flexible regions (Fig 1B) which are consistent with the presence of the CL region and the pre-N domain in our construct. Nevertheless, unlike the typical bell-shaped curve of a well-folded, single-domain protein with a local maximum of about 1.1 at *qR*g ≈ 1.73 (Burger et al, 2016), the dimensionless Kratky profile of apo mGRP94 manifested a much higher maximum of 1.4 at a shifted *qRg* ≈ 2.3 (Fig 1B). Such an observation suggests that mGRP94, in its apo form, samples extended shape in solution. We then calculated the distance distribution function *P*(*r*) via indirect Fourier transform (FT). As shown in Fig 1C, the *P(r)* profile of apo mGRP94 demonstrated a maximum diameter (*D*_max_) of 188.9Å and exhibited a multimodal distribution with two major peaks at 40 and 80 Å respectively, further indicating that dimeric mGRP94 bears inherent conformational heterogeneity and could take on an extended conformation in solution. Of note, the *P(r)* profile for apo mGRP94 in this work is slightly different from that observed in previous studies (Krukenberg et al, 2009). Specifically, although the *D*_max_ obtained is similar in both studies, the multimodal distribution of *P(r)* is much more prominent in the current study, further reflecting the impact of the preserved CL region and the pre-N domain on the overall conformation of apo mGRP94.

Table S1. SAXS data analysis, model fitting, and software used.

**Figure S2.**
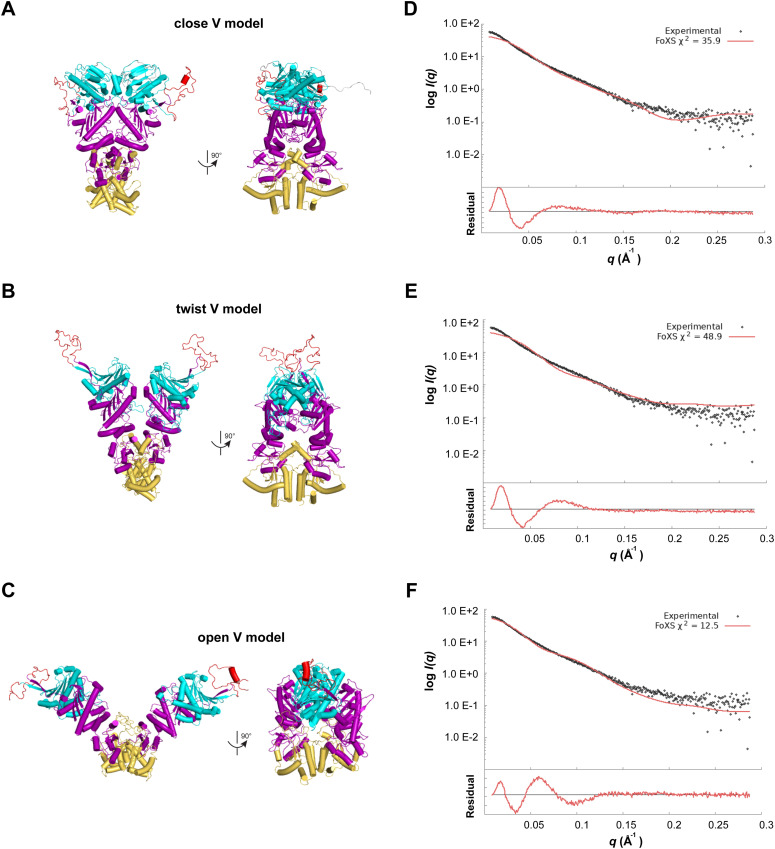
Comparison of theoretical and experimental scattering curves and residuals of the fit. **(A, B, C)** Cartoon representations of the mGRP94 “close V,” “twist V,” and “open V” models, with the pre-N (grey), N-terminal (cyan), charged linker (red), middle (purple), and C-terminal (orange) domains color-coded as in Fig S1A. **(D, E, F)** Theoretical scattering curves (red lines) of mGRP94 “close V” (D), “twist V” (E), and “open V” (F) models are overlayed with the experimental scattering data of the apo mGRP94 sample (gray dots). Residuals of the fit are shown at the bottom. Theoretical scattering curves are generated by FOXS (Schneidman-Duhovny et al, 2013).

Meanwhile, we modeled the structures of mGRP94 in “twist V” and “closed V” conformation based on the crystal structures of homologous (97% identity) dGRP94 (PDB: 2O1U and 5ULS), and the structure of mGRP94 in more extended “open V” conformation based on the crystal structure of apo *E.coli* HtpG (PDB: 2IOQ) (Fig S2A–C). Then, the theoretical scattering profiles of these structure models were simulated and compared with the experimental data (Fig S2D–F). The experimental scattering and the derived *P(r)* function of apo mGRP94 deviates significantly from the simulated scattering data of the individual models (Figs 1C and S2D–F), and the experimental *D*_max_ is also considerably larger than the models of mGRP94 in either “twist V” or “closed V” conformation. Notably, the deviation between the experimental scattering and the simulated scattering data is much smaller for “open V” model (χ^2^ = 12.5) than for “twist V” (χ^2^ = 48.9) or “close V” (χ^2^ = 35.9) model (Fig S2D–F), also suggesting that “open V”-like extended conformation likely exist in the apo mGRP94 population.

Together, these results indicate that apo mGRP94 adopts neither “twist V” nor “closed V” conformation as observed in crystal structures, but rather exists as a heterogeneous population wherein extended and compact conformations are likely both present.

### “Extended open V,” “compact close V,” and “compact twist V” conformations of apo mGRP94 coexist in the solution

Previous SAXS studies have shown that cytosolic hsp90s in their apo form appear to be heterogenous and extended in solution, with the *D*_max_ for HtpG, Hsc82, pig Hsp90, and human Hsp90α being 170, 220, 195, and 200 Å respectively, all of which are close to the *D*_max_ of mGRP94 obtained in this study (Krukenberg et al, 2008; Krukenberg et al, 2009). Notably, the shape of their *P(r)* functions are also quite similar to each other and the experimental *P(r)* profile of mGRP94 from this study, wherein they all manifest a multimodal and elongated distribution, indicating coexistence of multiple conformations (Krukenberg et al, 2009).

**Figure 2.**
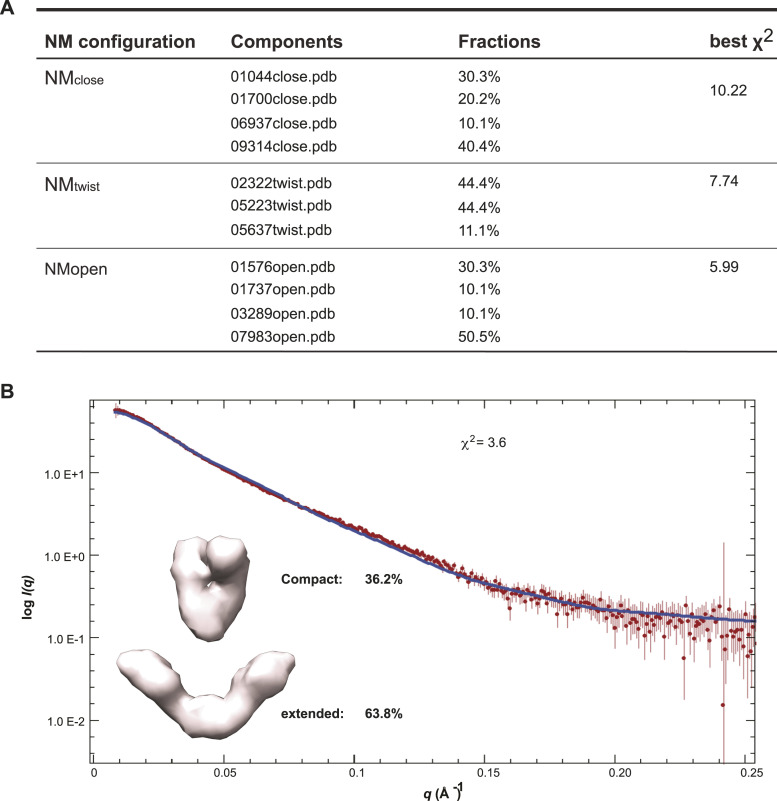
“Extended” and “Compact” conformations of apo mGRP94 coexist in solution. **(A)** Ensemble modeling results for apo mGRP4. **(B)** The fitting (blue line) of the OLIGOMER-picked models (inset) to the experimental profiles (red squares) is shown. Note that the models were rendered at 30 Å because the fit to the experimental SAXS data is good up to *q* = 0.05 Å^−1^, suggesting that the models are reliable up to a resolution level at about 20 Å.

To quantitatively account for the conformational heterogeneity of apo mGRP94 in solution, we then used the Ensemble Optimization Method (EOM) to look for all possible conformations of it. During the ensembles generating process of EOM, the NTD and MD domains of mGRP94 were treated as a rigid body (NM domain) to speed up the conformational space searching, and the orientation of the two NM domains relative to the dimerized CTD domain was allowed to vary in a twofold symmetric manner. We first fixed the relative configuration of the NTD domain to the MD domain as observed in the “closed V” conformation of dGPR94, EOM analysis using two such NM_closed_ domains and one dimerized CTD domain resulted in a moderate fit (χ^2^ = 10.22) to the experimental SAXS data (Fig 2A) while substituting the NM configuration for that observed in the “twist V” conformation of dGRP94 (NM_twist_) leads to a better fit at χ^2^ = 7.74 (Fig 2A). Among the cytosolic hsp90s, only *E.coli* HtpG has an available crystal structure in apo form, which manifests an “open V” conformation with its two N domains 150 Å apart from each other (Shiau et al, 2006). We thus also modeled an “open V” conformation of mGRP94 based on this crystal structure and then used its NM configuration (NM_open_) for EOM analysis. The use of NM_open_ further improved the χ^2^ to 5.99 (Fig 2A), but the fit is still far from ideal.

To further improve the fit, we then used OLIGOMER to account for the coexistence of distinct conformations. The input for OLIGOMER is the scattering profiles of apo mGRP94 and the form factors of mGRP94 models in “closed V” and “twist V” conformations, and that of the best EOM models (i.e., 01576open.pdb, 01737open.pdb, 03289open.pdb, and 07983open.pdb from previous EOM analysis). Given the simulated scattering intensities from the various models fed in, OLIGOMER finds their volume fractions by solving a system of linear equations using the algorithm of unconstrained least-squares to minimize the discrepancy between the experimental and calculated scattering curves. In this case, the OLIGOMER analysis resulted in a further improved fit to the experimental data (χ2 = 3.6) (Fig 2B) and revealed the presence of at least two distinct conformational states (Fig 2B). Most of mGRP94 (63.8%) samples an extended conformation which resembles the “open V” configuration of apo *E.coli* HtpG, whereas the rest (36.2%) seems to take on a compact shape (Fig 2B, inset). Hence, consistent with the large *D*_max_ and the multimodal-shaped *P(r)* profile, EOM and OLIGOMER analysis further support that apo mGRP94, akin to the cytosolic hsp90s, bears conformational heterogeneity in solution wherein the majority adopts extended conformation.

**Figure 3.**
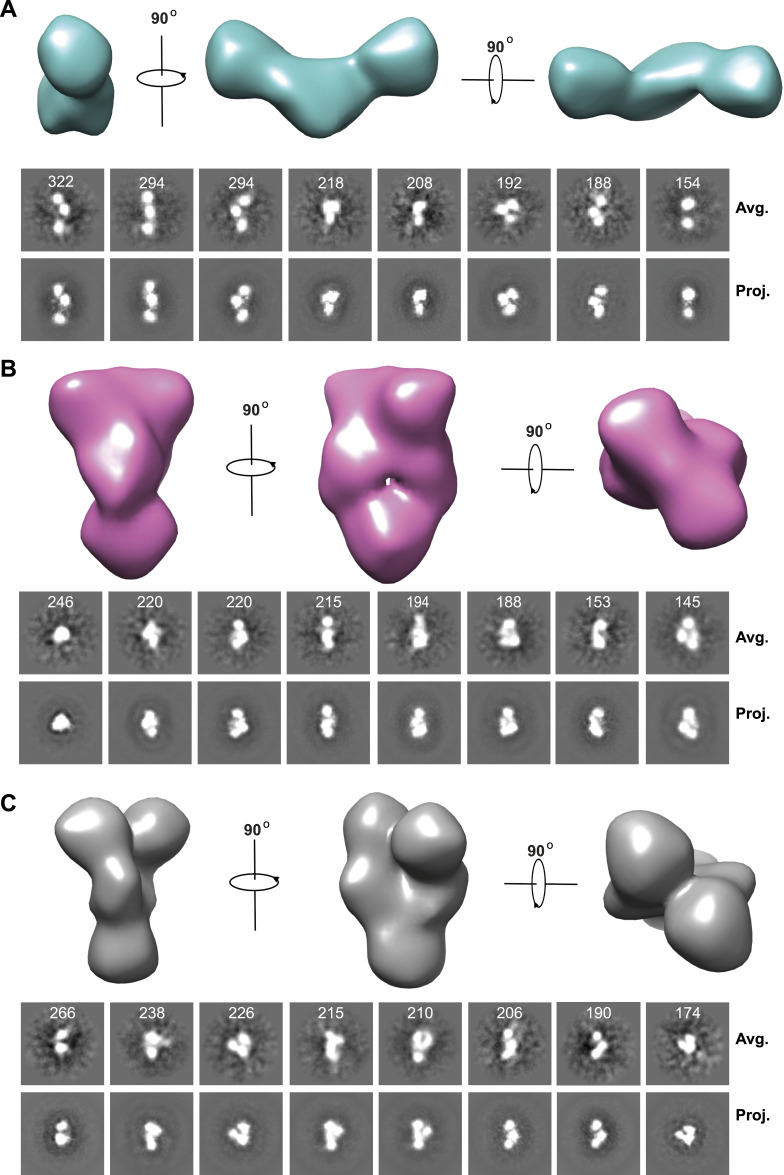
nsEM visualization of apo mGRP94 indicates the coexistence of “extended open,” “compact twist,” and “compact closed” conformations. **(A, B, C)** The nsEM reconstructions of apo mGRP94 unravel the coexistence of three different conformations. The nsEM reconstructions were shown in different views (top). To examine the correspondence between nsEM raw images and the final reconstructions, reference-free averages were compared with representative 2D projections of the final reconstructions at different angles. The number of particles included in each average is shown.

To gain more intuitive perception of the conformational heterogeneity of mGRP94, we next used negative staining EM (nsEM) to characterize mGRP94 in its apo form. Consistent with the SAXS results, apo mGRP94 manifested a high degree of conformational variability under the microscope, as molecules in either extended or curled-up conformation are readily discernable in reference-free 2D class averages. An initial dataset of 33,345 well-defined particles was used for ab initio model reconstruction without imposing any symmetry, whereas C2 symmetry was applied during subsequent 3D classification and 3D auto-refine. Finally, good reconstructions of three distinct classes were obtained (Fig 3), of which, the 2D projections match very well to corresponding reference-free class averages (Fig 3A–C, bottom panel).

**Figure S3.**
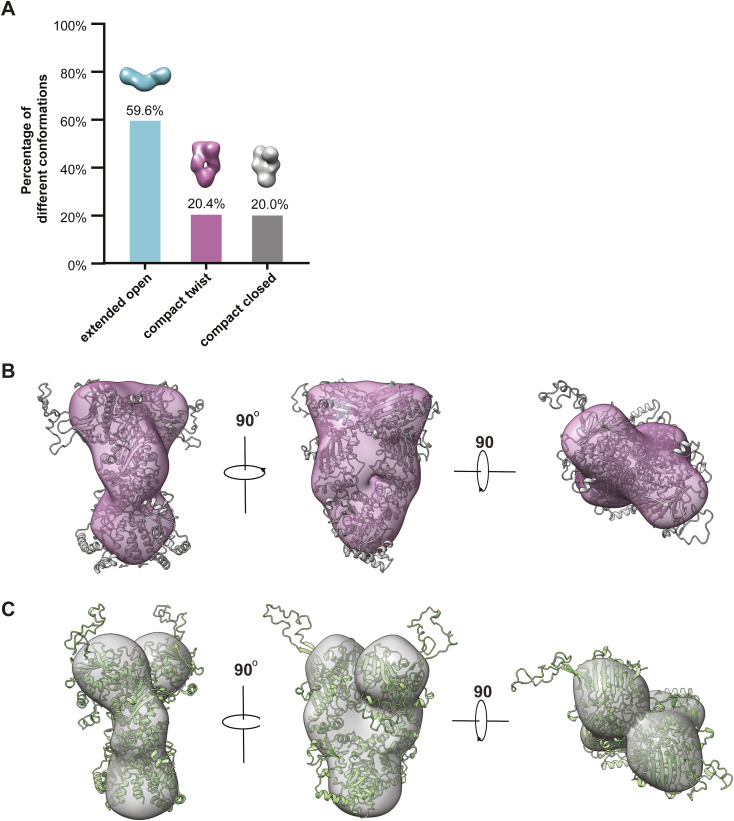
Relative abundancy and model fitting of nsEM reference-free 3D classes. **(A)** The frequency distributions of the three different conformations. **(B, C)** The class 2 and class 3 of nsEM reference-free 3D classes could fit well by the “close V” (B) and “twist V” (C)-like mGRP94 models.

The first class corresponds to about 59.6% of the particles’ entire populations (Fig S3A) and manifests an extended shape that resembles the “open V” conformation seen in the models that fit the SAXS data (Fig 3A, compared with 01576open.pdb in Fig 2B inset). The second class and the third class each correspond to 20.4% and 20% of the particle’s population (Fig S3A). Although class 2 and class 3 look similar at a cursory level, they are actually different from each other because the two parts which correspond to the two N terminal domains of mGRP94 separate from each other in class 3 yet contact each other in class 2 (Fig 3B and C). Indeed, class 2 and class 3 each could be fit well by the “close V” and “twist V” like mGRP94 models, and the resulting coefficients are 0.782 and 0.817, respectively (Fig S3B and C).

Together, the SAXS and nsEM results corroborate each other and converge to the same point that distinct conformations of mGRP94 coexist in solution in the absence of nucleotides, with the majority (∼60%) of apo mGRP94 samples an “extended open V” conformation, whereas the rest (∼40%) resides in either “close V” or “twist V”-like compact conformations.

### Impact of nucleotides on the conformational equilibrium of mGRP94

**Figure S4.**
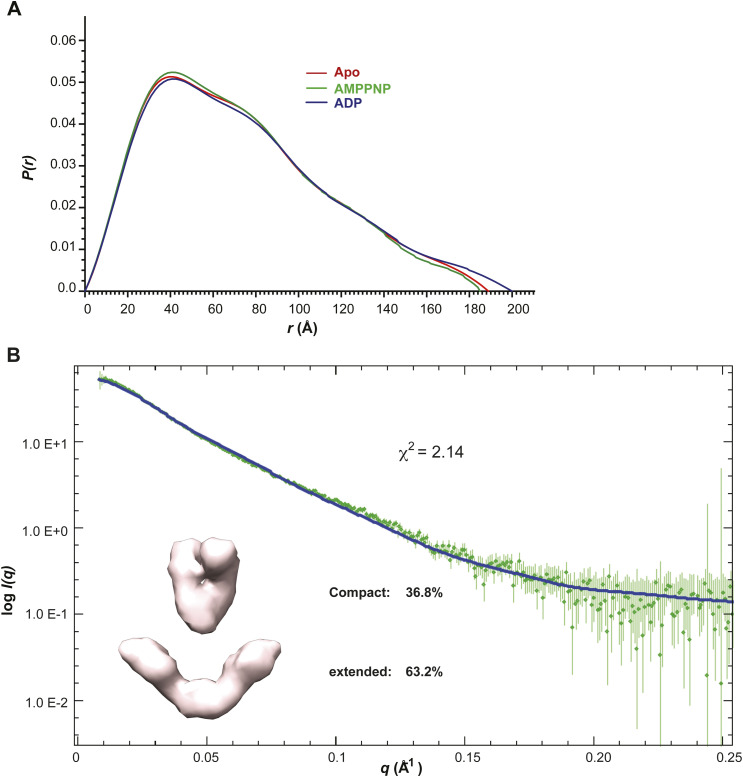
The binding of AMPPNP did not change the relative abundance of extended and compact conformations of mGRP94. **(A)** Normalized experimental *P(r)* curve of AMPPNP-bound mGRP94 (green) and ADP-bound mGRP94 (blue) are compared with that of apo mGRP94 (red). The *P(r)* curves are normalized to equal areas. Note that the presence of nucleotides caused barely noticeable shift in the *P(r)* profiles of mGRP94. **(B)** The fitting (blue line) of the OLIGOMER-picked models (inset) to the experimental profiles of AMPPNP-bound mGRP94 (green squares) is shown.

Although previous SAXS, nsEM, and single-molecule FRET investigations on *E.coli* HtpG and *S.cerevisia*e Hsc82 revealed a dynamic equilibrium of different conformational states that could be clearly shifted by the presence of nucleotides (Krukenberg et al, 2008; Southworth & Agard, 2008), the impact of nucleotides on the equilibrium is minimal in the case of mammalian Hsp90α (Krukenberg et al, 2009). To assess the effect of nucleotide on the conformational equilibrium of mGRP94, we collected SEC-SAXS data of it in the presence of saturating ADP or AMPPNP (Fig S1E and F). Similar to Hsp90α but different from HtpG or Hsc82 (Krukenberg et al, 2009), the addition of AMPPNP caused a barely noticeable shift in the *P(r)* profiles of mGRP94 (Fig S4A). The *D*_max_ of AMPPNP-bound mGRP94 is 185.4 Å, largely the same as that of apo mGRP94 (Table S1), although there appears to be a small rise in the probability of paired distance between 40–60 Å and a small decrease at longer distances (>140 Å) (Fig S4A). Consistently, scattering data of AMPPNP-bound mGRP94 is best fit by 63.2% extended and 36.8% compact conformation (Fig S4B), which is very similar to the fit of apo mGRP94 (63.8% extended and 36.2% compact). Likewise, the addition of ADP did not cause obvious shifts in the *P(r)* profiles of mGRP94 except for a small increase at longer distances (>160 Å) (Fig S4A).

**Figure 4.**
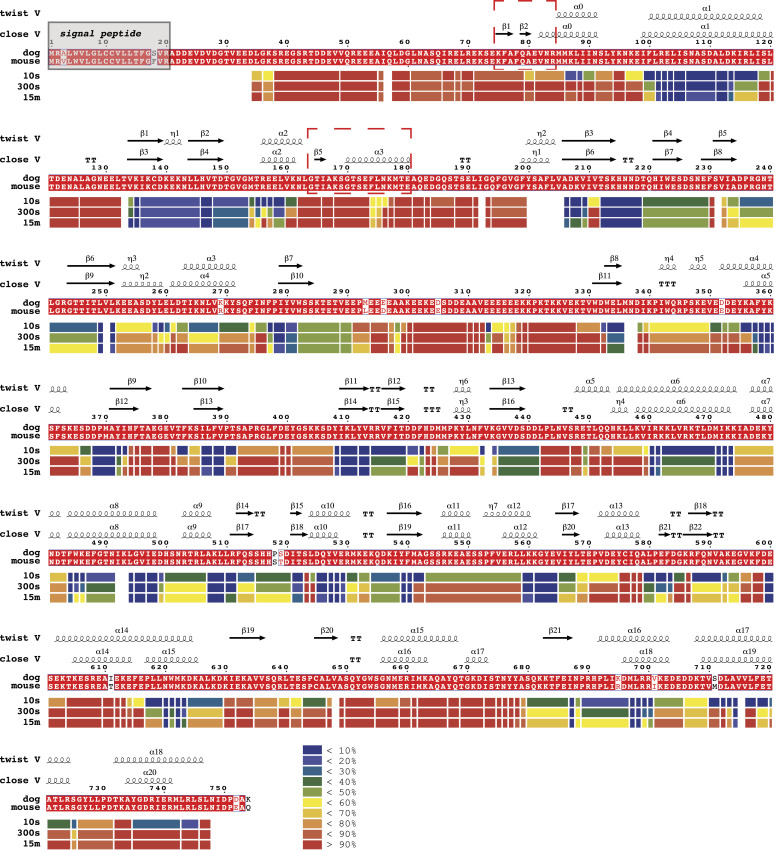
In-solution conformational dynamics of apo mGRP94 as revealed by HDX. The HDX profile of apo mGRP94 at different time points is presented as a heatmap under the aligned sequence of mGRP94 and dGRP94 (sequence identity: 97%). The inset shows the color coding for different percentages of deuteron incorporation. Above the alignments, secondary structural elements of dGRP94 in “compact twist V” and “compact close V” structures are shown. Note that the regions in mGRP94 that manifest low HDX rate generally match dGRP94 regions that adopt α-helix or β-sheet conformation in both structures, whereas the regions in mGRP94 that exhibit high HDX rate usually corresponds to dGRP94 regions that are consistently flexible in both structures. Notably, 75–84 aa and 165–180 aa (red boxes) adopt a folded conformation in a “compact close V” structure yet appear disordered in a “compact twist V” structure, and thus are chosen as “indicative regions” to signal the conformational transitions of mGRP94 between the two structures.

**Figure 5.**
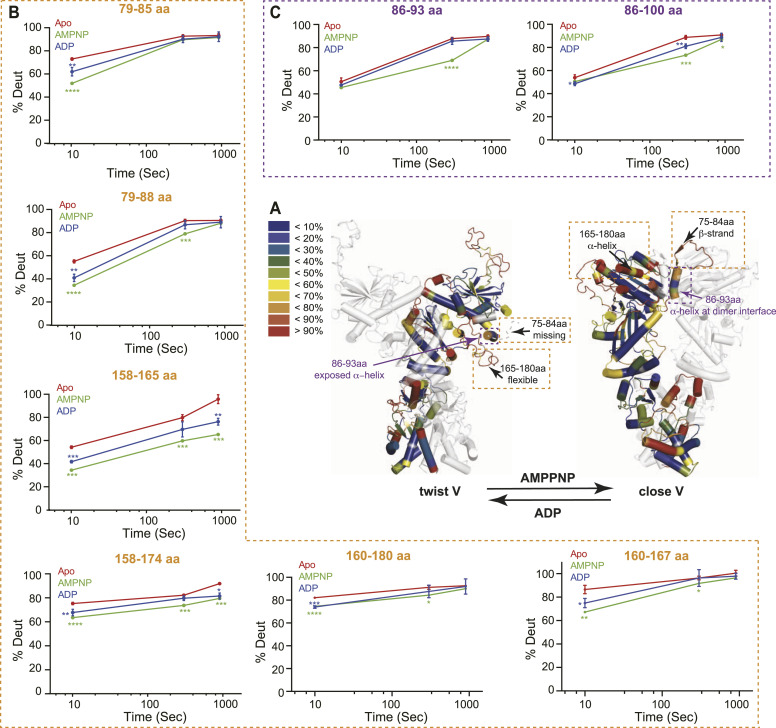
The conformational transition of mGRP94 in the presence of AMPPNP or ADP as captured by HDX-MS. **(A)** Deuteron incorporation at 10 s for apo mGRP94 was mapped onto one protomer of the “twist V” and “close V” models, respectively, the other protomers in these models were colored grey and rendered transparent for clarity. The inset shows the color coding for different percentages of deuteron incorporation. The black arrows and the orange boxes indicate the position of the indicative regions in different models. The purple arrows indicate the positions of 86–93 aa (purple boxes) in different models. The conformations of the indicative regions and 86–93 aa in different models are also labeled. **(B, C)** The deuterium uptake data of each indicated peptide is plotted as percent deuterium uptake versus time on a logarithmic scale. **(B, C)** Binding of AMPPNP (compare green lines with red lines) induced obvious HDX protection in “indicative regions” (B) and regions encompassing 86–93 aa (C), although with different kinetics. In “indicative regions,” the HDX protection effect appears as early as 10 s, whereas in regions ranging from 86–93 aa, the HDX protection effect is prominent at 300 s. In the presence of ADP (blue lines), the HDX of 86–93 aa is close to that in the apo state (red lines), whereas the HDX of indicative regions is faster than that in the AMPPNP-bound state (green lines), but still slower than that in the apo state (red lines). The *P*-values were given by *t* test, comparing the #D of the same peptide in AMPPNP or ADP state against the apo state. ****P < 0.0001, ***P < 0.001, **P < 0.01, and *P < 0.05.

The SAXS data thus suggest a minimum impact by AMPPNP or ADP on the relative abundance of “extended” versus “compact” conformations. Nevertheless, SAXS profiles generally offer low resolution (∼20 Å) information on the overall shape of macromolecules and would fail to inform if the conformational differences are not as prominent as that between “extended” and “compact” conformations but akin to the difference between “compact close V” and “compact twist V” conformations. To investigate this hypothesis, we propose to characterize the in-solution dynamic conformations of mGRP94 using HDX-MS, considering that HDX-MS could provide conformational information of macromolecules at higher resolution, that is, peptide level. Previously, nucleotide-bound dGRP94 has been captured in either “compact twist V” (PDBID: 2O1U and 2O1V for AMPPNP- and ADP-bound dGRP94 respectively) or “compact close V” (PDBID: 5ULS, AMPPNP-bound dGRP94) conformations. Comparing these structures, regions 75–84 aa and 165–180 aa caught our attention as they adopt completely different shapes in these two conformations (Fig 4, red boxes and Fig 5A, black arrows). Although completely disordered (and thus unseen) in the “compact twist V” conformation, residues 75–80 aa folded into consecutive β-strands and crossed to the opposite protomer to contribute in NTD dimerization in the “compact close V” structure of dGRP94 (Dollins et al, 2007; Huck et al, 2017). Meanwhile, residues 81–84 aa and residues 165–180 aa each folded into α-helices in the “compact close V” conformation but appear disordered (and thus invisible) in the “compact twist V” conformation. Considering that well-folded and disordered regions will exhibit completely different HDX rates in solution, we reasoned that the HDX profiles of these regions (hereafter referred to as “indicative regions”) could be leveraged to indicate the in-solution transition between “compact close V” and “compact twist V” conformations.

### The nucleotide-loading state dictates the conformational equilibrium of mGRP94 between “compact twist V” and “compact close V” structures

**Figure S5.**
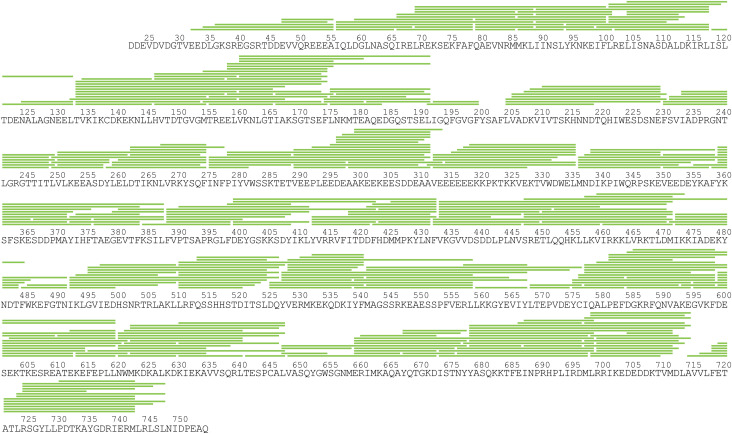
The HDX-MS peptide coverage map for mGRP94. Green lines above the protein sequence represent the digested peptides that were identified and analyzed in this study.

We first monitored the deuterium exchange of apo mGRP94 and obtained excellent peptide coverage (Fig S5). Consistent with the high sequence identity (97%) between mGRP94 and dGRP94, the in-solution HDX heatmap of apo mGRP94 matches the secondary structure profile of dGRP94 well (Fig 4, compare the HDX heatmap to the secondary structural elements listed above the alignment), wherein regions with well-defined secondary structures generally manifest low to medium HDX rate at 10 s and vice versa.

**Figure S6.**
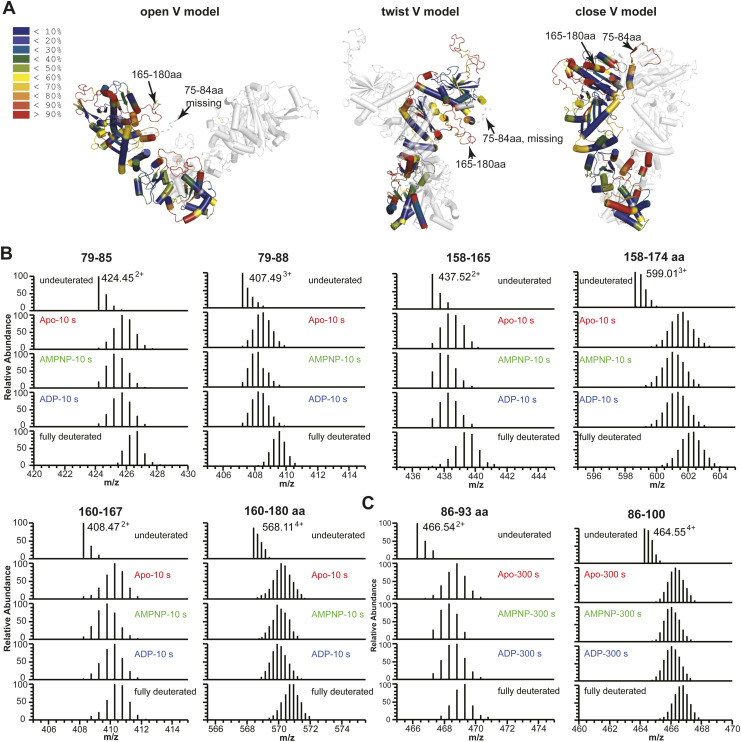
Mass spectra of peptides showing HDX protection in the presence of AMPPNP and ADP. **(A)** Deuteron incorporation at 10 s for apo mGRP94, mapped onto one protomer of the “open V,” “twist V,” and “close V” models, respectively, the other protomers in these models were colored grey and rendered transparent for clarity. The inset shows the color coding for different percentages of deuteron incorporation. The arrows indicate the position of the indicative regions in different models. **(B, C)** Mass spectra of indicated peptides from mGRP94 in different nucleotide-bound states are compared with each other in parallel (second to fourth panel), with the mass spectra of undeuterated and fully-deuterated samples shown as controls at the top and bottom panels. **(B, C)** Mass spectra of all identified peptides from the “indicative regions” (B) and mass spectra of peptides from the regions ranging 86–93 aa (C) are shown.

We then focused on the “indicative regions” (Fig 4, red boxes and Fig 5A, black arrows). In apo mGPR94, the “indicative regions” manifest high levels of deuterium incorporation within 10 s, indicating that disordered conformation dominates these regions in the absence of nucleotides (Fig 4, red boxes and Fig 5A, black arrows). Given the relative abundance of “open V,” “twist V,” and “closed V” conformations in apo mGRP94, such a result suggests that these “indicative regions” take on similar disordered conformation in “open V” conformation as seen in “twist V” conformation (Fig S6A, arrows). Obvious protection from HDX, that is, HDX rate decrease, was observed for regions encompassing 75–84 aa and 165–180 aa upon the addition of AMPPNP (Figs 5B and S6B, and Table 1), indicating a conformational transition from unfolded to folded state. As mentioned earlier, 75–84 aa and 165–180 aa appear well-folded in the “close V” structure of dGRP94 (Fig 5A), which would predict a low HDX rate. Thus, the observed HDX decrease in the “indicative regions” upon AMPPNP addition is consistent with a conformational transition of “compact” mGRP94 from “twist V” to “close V” structure.

**Table 1.** HDX data summary.

Data Set	GRP94	GRP94-ADP	GRP94-AMPNP
GRP94	1 μM	1 μM	1 μM
Ligand(ADP/AMPNP)	0 μM	20 μM	20 μM
HDX reaction details	50 mM Tris, pH 8.0, 150 mM NaCl, 10°C		
HDX time course	0, 10, 300, and 900 s		
HDX control samples	non-deuterated and fully deuterated controls (GRP94)		
Back-exchange (mean/IQR)	39.51%/19.81%		
# of peptides	405		
Sequence coverage	94%		
Average peptide length/redundancy	18.10/8.00		
Replicates (biological or technical)	3 (biological)		
Repeatability	0.027 (average SD)	0.074 (average SD)	0.056 (average SD)
Significant differences in HDX (ΔHDX > X D)	95% CI		

Besides the “indicative regions”, protection from HDX was also prominent for residues ranging 86–93 aa upon AMPPNP addition (Figs 5C and S6C and Table 1). Different from the high basal HDX rate of “indicative regions,” the region ranging 86–93 aa manifested a low HDX rate at 10 s in apo mGRP94, and AMPPNP-induced HDX protection of this region is most prominent at the time point of 300 s (Figs 5C and S6C and Table 1). Notably, 86–91 aa appears as α-helix in both “close V” and “twist V” structures, indicating its inherent α-helical propensity that is consistent with the observed low HDX rate of 86–93 aa region at 10 s. Nevertheless, although 86–91 aa appears as α-helix in both structures (Fig 5A, purple arrows), the environment surrounding it is completely different in these two situations. In the “twist V” structure, the 86–91 aa α-helix remains exposed to the solvent and makes very limited interactions with other parts of NTD; whereas in the “close V” structure, the same region becomes buried at the homodimer interface and makes extensive interaction with the NTD and MD from the other protomer (Fig 5A, purple arrows) (Dollins et al, 2007; Huck et al, 2017). Hence, the observed AMPPNP-induced HDX protection of 86–93 aa region is also consistent with a “twist V” to “close V” conformational transition of mGRP94 as such transition would change its local environment and consequently shield this region from HDX (Figs 5A and S6A). Together, the SAXS and HDX-MS results support a model wherein the binding of AMPPNP to mGRP94 would not shift the equilibrium between its “extended” and “compact” conformations but rather render “compact” mGRP94 to undergo a “twist V” to “close V” conformational transition.

Next, we compared the HDX profile of ADP-bound mGRP94 with that of AMPPNP-bound or apo mGRP94 to look for the potential impact of ATP hydrolysis. In the presence of ADP, the HDX levels of 86–93 aa are much higher than their HDX levels in the AMPPNP-bound state, and close to their HDX levels in apo mGRP94 (Figs 5C and S6C, 86–93 aa). Such observation suggests a “close V” to “twist V” back transition upon the hydrolysis of ATP into ADP. Different from this region, the HDX of 158–165 aa region in the ADP-bound state is faster than that in the AMPPNP-bound state, but still obviously slower than that in the apo mGRP94 (Figs 5B and S6B, 158–165 aa). Whereas the elevated HDX rate in ADP-bound state (as compared with AMPPNP-bound state) is consistent with the “close V” to “twist V” back-transition model, the residual HDX protection rendered by ADP (as compared with apo mGRP94) suggests that some residues within 158–165 aa may be directly or indirectly involved in nucleotides engagement and thus can only regain their full HDX potential when nucleotides are released. Indeed, 158–165 aa constitutes one side of the nucleotides binding pocket and a shielding effect on it is thus assumed in the presence of either AMPPNP or ADP (Immormino et al, 2004; Dollins et al, 2005).

## Discussion

The structural dynamics of proteins are pivotal for their functions, and characterizing such dynamics is often indispensable for understanding their mechanism of action and regulation. SAXS and HDX-MS are both solution techniques well suited to probe protein dynamics (Wales & Engen, 2006; Jacques & Trewhella, 2010). Whereas SAXS generally provides a low-resolution overall dynamic shape of a protein, HDX-MS could instead offer sensitive information on conformational dynamics at peptide level (Englander, 2006). As such, information gained by these two techniques often complements each other and together they could hint at the functional movements of a protein during action. Nevertheless, both techniques measure the protein of interest in bulk mode, therefore the final information given by them is an average of all the protein molecules in a population. For proteins that manifest conformational heterogeneity, for example, mGRP94 in this study, correct interpretation of SAXS and HDX-MS data needs the assistance of other tools, such as nsEM or cryoEM, which could help to clarify what exists in the population through capturing and sorting the images of each individual in the population (Engen & Komives, 2020).

Guided by these considerations, here we combine nsEM, SAXS, and HDX-MS to probe the conformational dynamics of mGRP94 along its ATP hydrolysis cycle. Processed SAXS data clearly indicate that apo mGRP94 exists as a heterogenous population wherein extended conformations are dominant (63.8%) (Figs 1 and 2). Subsequent nsEM visualization of apo mGRP94 further confirmed the coexistence of one extended “open V” and two compact conformations in the heterogenous population (Fig 3), wherein the two compact conformations are well accounted by the previously captured “twist V” and “close V” structures, respectively (Dollins et al, 2007; Huck et al, 2017). Consistent with earlier observations (Krukenberg et al, 2009), adding AMPPNP or ADP to the system did not produce noticeable changes in the SAXS profile (Fig S4), suggesting that the equilibrium between “extended” and “compact” conformations may not change along the ATP hydrolysis cycle of mGRP94. We then used HDX-MS to further characterize the dynamics of mGRP94 in the presence of AMPPNP or ADP, reasoning that the higher sensitivity and resolution of HDX-MS would help to inform on subtle conformational changes that fail to produce a signal in SAXS profiles. Indeed, using HDX-MS, we successfully visualized the AMPPNP-binding induced “twist V” to “close V” conformational transition of mGRP94 and a “close V” to “twist V” back transition when ADP replaces AMPPNP (Figs 4 and 5). Collectively, these observations also reconciled the long-lasting discrepancies between crystallography snapshots and SAXS measurements of AMPPNP-bound mGRP94, wherein the snapshots captured two distinct compact conformations of mGRP94, whereas SAXS data suggest the dominating status of an extended conformation instead. Notably, the impact of nucleotide on GRP94 is different from that on HtpG and hsc82, two other well-studied Hsp90 paralogs. Whereas the presence of nucleotides clearly shifts the equilibrium between the “extended” and “compact” conformations of HtpG and hsc82 (Southworth & Agard, 2008; Krukenberg et al, 2009), the presence of nucleotides only drives the transition between the two compact conformations of GRP94 while leaving the extended population unaffected.

A limitation of the current study lies in the less-accurate quantification of the relative abundance of the “extended open V,” “compact twist V,” and “compact close V” conformations. On one hand, quantitative fitting of the SAXS results could only discriminate between “extended” and “compact” conformations; On the other, although nsEM captured the coexistence of two “compact” conformations, that is, “close V” and “twist V,” the relative abundance of each conformation given by nsEM might be not accurate, as that can be influenced by the data processing process. To this end, a recently released computational approach might be repurposed to provide a more accurate appreciation of the relative abundance of the three conformations according to the experimental HDX-MS data (Bradshaw et al, 2020).

Here, we revisited the in-solution dynamics of GRP94 along the ATP cycle and provided information for future investigations of how the conformational changes of GRP94 are relayed to the folding of its clients. The fact that the binding and hydrolysis of ATP only drives the transition between the “compact twist V” and “compact close V” conformations renders the coexistence of the highly abundant (∼60%) “extended open V” conformation intriguing. One viable explanation for the usefulness of the “extended open V” conformation would be that such conformation, given its bigger exposed surface between the two protomers, can better attract and accommodate large clientele of GPR94. It is also possible that the energy barrier between the “extended open V” and “compact twist V” conformations of mGRP94 is not high in the absence of clientele, thus GPR94 can transit back and forth between these two conformations to better trap clientele of different sizes. Once the clientele is bound, GRP94 would have a higher chance to populate the “compact twist V” and prepare for its transition into the “compact close V” conformation upon the binding of ATP and co-chaperons like Bip (Huang et al, 2022). We also envision that the integrative interpretation of X-ray crystallography, SAXS, nsEM, and HDX-MS data, as illustrated in this article, may also be applicable in studying other complex biological systems that exhibit similar structural flexibility and heterogeneity in solution.

## Materials and Methods

### Gene cloning, protein expression, and purification

The gene of mouse GRP94(22–754) was cloned into a modified pST39 vector fused with an N-terminal Strep-tag Ⅱ followed by a thrombin cleavage site and a C-terminal tobacco etch virus protease cleavage site followed by a 6xHis tag.

The recombinant plasmid pST39-GRP94 was transformed into *Escherichia coli* BL21 (DE3) cells for protein expression. The cells were cultured in Luria–Bertani (LB) medium at 37°C until the OD_600_ reached 0.8. The cultures were then incubated for 16–20 h at 14°C in the presence of 0.2 mM isopropylthio-β-D-galactoside (IPTG) before being harvested by centrifugation at 5,000*g* for 5 min. The cells were then resuspended in buffer A (50 mM Tris–HCl, pH 8.0, 200 mM NaCl, 10% glycerol, 5 mM β-Me) supplemented with 20 mM imidazole, lysed by sonication, and clarified by centrifugation at 70,000*g* for 60 min. The supernatant was loaded onto a Histrap HP column (GE Healthcare). Elution fractions from the Histrap column were then pooled, cleaved by tobacco etch virus protease, and further purified on a Hitrap Q HP column (GE Healthcare) with a linear gradient of 50–500 mM NaCl. Elution fractions from the Hitrap Q HP column were then passed through the Histrap HP column again and GRP94 proteins in the flow through were then pooled and further purified with a Superdex S200 column (GE Healthcare) equilibrated with buffer A. Peak fractions from the Superdex s200 column were pooled, concentrated and stored at −80°C until further use.

### SEC-SAXS data collection and analysis

SAXS experiments were performed in SEC-SAXS mode at beamline BL19U2 of the National Facility for Protein Science Shanghai at the Shanghai Synchrotron Radiation Facility. The wavelength, λ, of X-ray radiation was set as 1.03 Å. Scattered X-ray intensities were collected using a Pilatus 1M detector (DECTRIS Ltd). The sample-to-detector distance was set as 2.76 m such that the detecting range of momentum transfer (*q* = 4π sinθ/λ, where 2θ is the scattering angle) was 0.009–0.29 Å^−1^. For SEC-SAXS, the buffer used was 25 mM HEPES, pH 8.0, 200 mM NaCl, and 1 mM TCEP with or without 1 mM ADP or AMPPNP. SEC-HPLC was performed on the WYATT SEC column (WTC030N5; WYATT Inc). The inline SEC-SAXS system (Liu et al, 2018) was operated at 0.50 ml/min flow rate. The columns and the SAXS flow cell were maintained at 25°C. The mGrp94 protein concentration was 10 mg/ml in the same running buffer, and 100 μl of the sample was injected into the column after centri-filtration. The 2-D scattering images were converted to 1-D SAXS curves through azimuthally averaging after solid angle correction and then normalizing with the intensity of the transmitted x-ray beam, using the software package BioXTAS RAW (Hopkins et al, 2017). Background scattering was subtracted using PRIMUS in the ATSAS software package (Petoukhov et al, 2012). Linear Guinier plots in the Guinier region (q*R_*g*_ < 1.3) were confirmed in all experimental groups. Pair distance distribution functions of the particles P(r) and the maximum sizes D_max_ were computed using GNOM (Svergun, 1992). Structural parameters from SEC-SAXS were summarized and compared in Table S1.

The volume fractions of coexisted conformations in solution were computed from scattering data using *OLIGOMER* (Konarev et al, 2003). The experimental scattering profile is considered as a mixture of several components and a set of form factors for each component of the mixture is built. Given the intensities from the components (form-factors), *OLIGOMER* finds the volume fractions by solving a system of linear equations using the algorithm of non-negative or unconstrained least-squares to minimize the χ^2^ values between the experimental and calculated scattering curves. The distinct conformations of mGRP94 were calculated and presented.

### EOM

EOM was implemented by the EOM 2.1 program (Tria et al, 2015). Starting from the atomic models of the respective NTD, MD, dimerized CTD domains, which were assembled with the available atomic models and treated as independent rigid bodies, and the orientation of the two NM domains relative to the dimerized CTD domain was allowed to vary in a twofold symmetric manner. A pool of 10,000 independent models based on sequence and high-resolution individual domains were first generated and we computed the theoretical scattering intensities of the models in the pool using a genetic algorithm for the selection of an ensemble. The averaged theoretical scattering intensity from independent ensembles was compared against that of the experimental scattering data. The ensemble that best describes the scattering data was selected.

### Homology model building and theoretical scattering profile simulation

Structural models of mGRP94 in “close V,” “twist V,” or “open V” conformations were derived by homology modeling using SWISS-MODEL (Waterhouse et al, 2018). Theoretical scattering curves of these models are generated by FOXS (Schneidman-Duhovny et al, 2013).

### HDX-MS

Amide hydrogen exchange of GRP94 alone was started by diluting 2 μl protein sample at 50 μM into 18 μl D_2_O buffer (50 mM Tris, pH 8.0, 150 mM NaCl) at 10°C. At different time points (0, 10, 300, and 900 s), the labeling reaction was quenched by the addition of chilled quench buffer (400 mM KH_2_PO4/K_2_PO4, pH 2.2, 50 mM TCEP) and immediately frozen in liquid nitrogen. For the HDX-MS of GRP94 in the presence of nucleotides, 1 μl GRP94 at 100 μM was first mixed with 1 μl AMPPNP or ADP at 2 mM. The mixture was then labeled by adding 18 μl D2O buffer before being quenched at different time points and flash-frozen. All frozen samples were stored at −80°C until analysis. For each HDX time point, three samples were prepared and analyzed. Fully deuterated samples were also prepared and analyzed to allow corrections for back-exchange.

The thawed samples were immediately injected into a HPLC-MS (Thermo ultimate 3000) system equipped with in-line peptic digestion and desalting. In-line digestion of injected samples was performed with an immobilized pepsin column (2 mm × 2 cm, house-packed). Digested peptides were then captured on a housed-packed POROS R2 (Thermo Fisher Scientific) trap column (1 mm × 2 cm), and desalted for 4 min. The desalted digests were separated using a Hypersil Gold C18 analytical column (1 mm × 50 mm; Thermo Fisher Scientific) over a 12 min gradient and directly analyzed with an Orbitrap Fusion mass spectrometer (Thermo Fisher Scientific) equipped with an Electrospray ionization source. The ion transfer tube was set at 220°C and spray voltage at 3.8 kV. Sample handling, protein digestion, and peptide separation were all conducted at 0–4°C to minimize back exchange. Mass spectrometry data were acquired in the range of m/z 350–1,400 for 18 min in positive mode. The HPLC system was extensively cleaned with blank injections between samples to minimize any carryover.

Peptide identification was performed by tandem MS/MS under orbi/orbi mode. All MS/MS spectra were analyzed using the MASCOT program, and final PSMs were filtered with a FDR of 1%. We carried out the initial analysis of the peptide centroids with HD-Examiner v2.3 (Sierra Analytics) and then manually verified every peptide to check retention time, charge state, m/z range, and the presence of overlapping peptides. The peptide coverage of GRP94 was found to be 94.0% and the relative deuteration levels (%D) of each peptide were automatically calculated by the HD-Examiner by normalizing against the fully deuterated sample. Key parameters from the HDX-MS assay were summarized in Table 1. The Table S2 stores the HDX data for kinetic analysis.

Table S2. HDX data table for kinetics experiments.

### Electron microscopy data collection and image processing

Grp94 protein samples (200 mM) in 50 mM Tris–HCl, pH 8.0, 200 mM NaCl, 10% glycerol, 5 mM β-Me were incubated for 10 min with or without 1 mM ADP or AMPPNP (Sigma-Aldrich) at 25°C. Grp94 protein was negatively stained by 0.75% uranyl formate on thin carbon-layered (10–20 nm thick) 400 mesh copper grids (EMCN) as previously described (Ohi et al, 2004). Negative-stained samples were imaged using a Talos C-Twin TEM (FEI) operated at 120 keV. Micrograph images were recorded using a 4 k × 4 k Ceta 16M camera (Thermo Fisher Scientific) at 720,00× magnification with 1.92 Å pixel size.

160 micrographs were recorded for the apo GRP94 dataset. Particles were automatically picked with crYOLO using a general mode (Wagner et al, 2019). Subsequent image processing was performed in cryoSPARC (Punjani et al, 2017). Selected particles were inspected, extracted from micrographs with a box size of 160 pixels, and subjected to reference-free 2D classification with a mask diameter of 240 Å. At least two rounds of 2D classifications were performed to select well-defined particle images. Ab initio reconstruction without imposing any symmetry was then launched, with the number of classes set to 4. Subsequent heterogeneous refinement (3D classification) was performed with C2 symmetry imposed using the four initial models obtained from the last step. After further 3D classification and 3D auto-refine, good reconstructions of three distinct classes were obtained from 17,083, 5,834, and 5,740 particles, respectively (Punjani et al, 2017). The reconstructed maps contoured to the approximate molecular weight of GRP94 were visualized in Chimera (Pettersen et al, 2004). The projections of refined 3D volumes were matched to experimental 2D class averages using the EMAN2 command e2classsvsproj.py (Tang et al, 2007).

## Data Availability

Further data supporting the findings of this study are available from the corresponding author upon reasonable request.

## Supplementary Material

Reviewer comments
